# Postnatal HIV transmission in breastfed infants of HIV-infected women on ART: a systematic review and meta-analysis

**DOI:** 10.7448/IAS.20.1.21251

**Published:** 2017-02-21

**Authors:** Stephanie Bispo, Lana Chikhungu, Nigel Rollins, Nandi Siegfried, Marie-Louise Newell

**Affiliations:** ^a^ Department of Social Statistics and Demography, University of Southampton, Southampton, UK; ^b^ School of Languages and Area Studies, University of Portsmouth, Portsmouth, UK; ^c^ Department of Maternal, New-born, Child and Adolescent Health, World Health Organisation, Geneva, Switzerland; ^d^ Medical Research Council, Cape Town, South Africa; ^e^ Human Development and Health, Faculty of Medicine, University of Southampton, Southampton, UK

**Keywords:** Antiretroviral therapy, HIV, prevention of mother-to-child transmission, breast feeding, systematic review, meta-analysis

## Abstract

**Introduction**: To systematically review the literature on mother-to-child transmission in breastfed infants whose mothers received antiretroviral therapy and support the process of updating the World Health Organization infant feeding guidelines in the context of HIV and ART.

**Methods**: We reviewed experimental and observational studies; exposure was maternal HIV antiretroviral therapy (and duration) and infant feeding modality; outcomes were overall and postnatal HIV transmission rates in the infant at 6, 9, 12 and 18 months. English literature from 2005 to 2015 was systematically searched in multiple electronic databases. Papers were analysed by narrative synthesis; data were pooled in random effects meta-analyses. Postnatal transmission was assessed from four to six weeks of life. Study quality was assessed using a modified Newcastle-Ottawa Scale (NOS) and GRADE.

**Results and discussion**: Eleven studies were identified, from 1439 citations and review of 72 abstracts. Heterogeneity in study methodology and pooled estimates was considerable. Overall pooled transmission rates at 6 months for breastfed infants with mothers on antiretroviral treatment (ART) was 3.54% (95% CI: 1.15–5.93%) and at 12 months 4.23% (95% CI: 2.97–5.49%). Postnatal transmission rates were 1.08 (95% CI: 0.32–1.85) at six and 2.93 (95% CI: 0.68–5.18) at 12 months. ART was mostly provided for PMTCT only and did not continue beyond six months postpartum. No study provided data on mixed feeding and transmission risk.

**Conclusions**: There is evidence of substantially reduced postnatal HIV transmission risk under the cover of maternal ART. However, transmission risk increased once PMTCT ART stopped at six months, which supports the current World Health Organization recommendations of life-long ART for all.

## Introduction

The annual number of new HIV infections in children has decreased substantially, from an estimated 520,000 in 2000 to 220,000 in 2014, representing a 58% decline [1]. This significant progress is consistent with improvements in the coverage and quality of antiretroviral treatment (ART) programmes providing prevention of mother-to-child transmission and treatment of HIV-positive adults [2,3].

HIV transmission from mother to child can occur during pregnancy, delivery, and breastfeeding [4]. Recognising the substantial benefit of breastfeeding for infant health and survival, and the need to minimise the risk of postnatal transmission through breastfeeding [5–7], the 2010 World Health Organization (WHO) guidelines recommended breastfeeding for infants of HIV-positive mothers for at least one year under the cover of maternal or infant ART [8].

In most African countries and some parts of India, health policy continues to advise mothers living with HIV to breastfeed [9,10]. In such settings, exclusive breastfeeding (EBF) for the first six months of life followed by complementary feeding and continued breastfeeding (CBF) for up to one year, under the cover of ART to either the mother or the infant [6] is recommended. However, these recommendations were drawn up supported by limited information on the risk of postnatal HIV transmission when women or child or both were on ART to prevent mother-to-child transmission (PMTCT). In addition, there was little or no information on whether mixed feeding (MF), which had been associated with increased risk of postnatal transmission in earlier studies, remained a risk even in the presence of ART [6,11].

In the past five years, further evidence has become available from studies and programmes where PMTCT postnatally was achieved through maternal ART or infant prophylaxis. The risk of transmission when infants receive other feeds in addition to breast milk in the first six months of life is of particular interest for public health programmes [12]. To this end, we present the results from a systematic review and GRADE Evidence summary tables, addressing the question of HIV transmission rates at six, nine, 12 and 18 months in infants born to women who were on ART from early-mid pregnancy until at least six months postpartum, and whose infants breastfed in the first six months of life or longer. This study was commissioned by the WHO and contributed to the formulation of the 2016 WHO HIV Infant Feeding guidelines [13].

## Methods

The review considered both experimental and observational studies, and included HIV- positive mothers receiving ART and their breastfed children regardless of receipt of infant antiretroviral prophylaxis. The exposures were HIV antiretroviral therapy and feeding modality during breastfeeding (EBF, MF, RF-replacement feeding) and outcome measures were overall and postnatal HIV transmission rate at six, nine, 12 and 18 months.

We conducted searches in multiple electronic databases including PubMed, MEDLINE, EMBASE, Cochrane Central Register of Controlled Trials, Web of Science, and CINAHL for articles published between 2005 and 2015. As triple combination ART regimen were only introduced in public health programmes in 2004, we restricted the selection of studies to be included in this review to publications from 2005. Reference lists from relevant studies, grey literature and conference abstracts available online from the International IAS AIDS Conference in Melbourne 2014 and the 2013-2015 Conferences on Retroviruses and Opportunistic Infections (CROI) were also searched.

**Figure 1.**
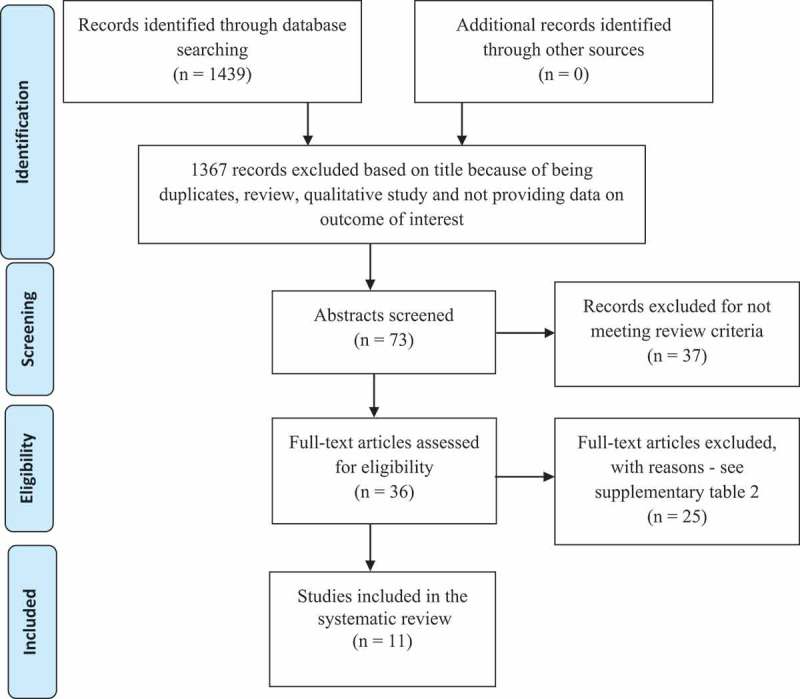
Flowchart of screening process.

Studies were first selected by SB and LC on the basis of eligibility from their abstracts. Full text of selected studies was then assessed with disagreements between the two reviewers resolved by a third reviewer (MLN). Data was extracted by SB and revised by LC. We have used the reporting standards described in the Preferred Reporting Items for Systematic Reviews and Meta-Analyses (PRISMA) statement [14], and the flow chart of the screening process is shown in Figure 1.

To obtain further information regarding infant feeding, we contacted first authors of seven studies and received responses from three of these, to solicit additional information regarding infant feeding modality in the first six months of life. Questions related to the type and duration of recommended feeding practice, type of infant feeding support provided to mothers, collection of data on child feeding practices, and how this data was addressed in the study (proportion, statistical model, rates of transmission or death).

## Assessment of study quality

A modified Newcastle-Ottawa Scale (NOS) was developed by the authors to assess the quality of all studies included in the analysis, on the basis of selection of study participants and outcome assessment [15]. Each study could score a maximum of five stars on Selection and four on Outcome; considering representativeness of the study population, ascertainment of exposure to ART and breastfeeding, report of maternal adherence to ART and feeding modality.

The information obtained from the NOS was used to comment on the quality of the included studies in the Grading of Recommendations Assessment, Development and Evaluation system (GRADE), a system for rating quality of evidence and grading strength of recommendations in systematic reviews [16]. In this study, GRADE considered study limitations, consistency of results, directness, imprecision and reporting bias.

Results are presented narratively and as pooled estimates with a heterogeneity score based on a random effects meta-analysis using STATA 13 (Stata Corp, 2013). Random effects were used because of methodological differences among studies, such as rates of EBF, incentives to keep treatment and time from which postnatal transmission was ascertained, what allows the true transmission rate to vary from study to study [17]. We summarised the information in graphs depicting overall (peripartum and postpartum transmission) and postnatal (transmission after negative HIV test at four to six weeks) HIV transmission rates at six and 12 months of age. Where no confidence interval was available for estimates from the study report, a confidence interval was calculated based on the number of events and those at risk using the formula described by Eayres (2008, [18]).

## Results

The search process identified 1439 citations, of which 1367 were excluded on the basis of being a duplicate, review, qualitative study or not evaluating transmission at six, nine, 12 or 18 months according to feeding modality (Figure 1). The abstracts of 72 studies were evaluated independently by three researchers (SB, LC and ML), and 11 studies were finally selected for inclusion in this review; four of these were cohorts nested within randomised clinical trials [11,19–21] and seven observational studies [5,22–27]. In all studies mothers started ART before or during pregnancy, and continued until at least six months postnatally, according to the WHO recommendations at the time. Seven studies followed this recommendation [5,11,19,20,22–24] with six-month ART, three provided lifelong ART for all women [21,25,26] while the remaining study [27] provided lifelong ART only for treatment-eligible mothers with very low CD4 count (see Supplementary Table 1 for more details on included studies).

**Table 1.** Responses regarding support for exclusive breastfeeding and type of data collection

	Studies	Duration of BF	Frequency counselling	Local of counselling	Type of support	Person giving support	Type of data collection
**Additional information**	Ngoma et al., 2015	12 months	Four weeks post enrolment, 36 weeks gestation, at birth, two weeks, six weeks, 3, 6, 9, 12, 15, 18 and 24 months	Two week was home visit, others facility based	Text message reminders, food and transportation stipends	Registered nurse	Interview
	Giuliano et al., 2013	4.5 months and wean over a 1.5 months	Every two weeks in first two months pregnancy and every month until BF cessation	Facility-based	Counselling	Skilled personnel in nutrition practices	Self-report
**Information from published papers**	Jamieson et al., 2012	Six months	1, 2, 4, 6, 8, 12, 18, 21, 24, 28 weeks post-partum	-	Counselling and breast-milk replacement food in case of BF cessation		Interview by standard questionnaire
	Alvarez-Uria et al., 2012	Six months	Not reported	Not reported	Not reported	Not reported	Not reported
	Coovadia et al., 2012	Six months	Seven days, 2, 5, 6, 8 weeks, 3, 6, 9, 12, 18 months	Not reported	Not reported	Not reported	Self-report
	Thomas et al., 2011	Six months	Weekly before delivery, and after delivery: 2, 6, 10, 14 weeks and 6, 9, 12, 15, 18, 24 months	Not reported	Not reported	Not reported	Not reported
	Marazzi et al., 2009	Six months	Not reported	Not reported	Counselling and nutritional supplement for pregnant and lactating mothers	Nutritionists	Clinical data
	Kilewo et al., 2009	Six months	1, 3, 6 weeks and 3, 4, 5, 6 months	Facility based	Counselling	Not reported	Not reported

## HIV transmission

Of the 11 studies, eight reported the transmission rate at age six months [5,11,19,20,22,23,25,26]. Two studies [19,26] did not provide a confidence interval around the estimated transmission rate, which was calculated using the formula [18]. Two studies reported the number of infections at six months and the number of children at risk: one study [26] was a retrospective cohort and provided a single number as a denominator (N = 856); and for the second study [19] the number of children at risk was obtained from the Kaplan-Meier graph. A further two studies reported the number of transmissions but not the number of children at risk [21,27] and one study reported transmission only at age nine months, but noted that all transmissions occurred during breastfeeding [24].

## Overall transmission at age six months

**Figure 2.**
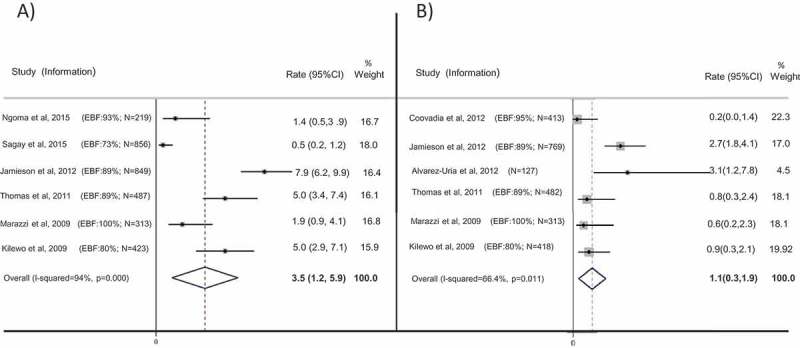
Transmission rates at age six months: (a) overall transmission rate (including peripartum) at age six months; (b) postnatal transmission rate between four and six weeks of age and age six months, with 95% confidence intervals, in children who were breastfed and whose mothers were on ART. Note: Red dashed lines represent final pooled estimate for overall (a) and postnatal (b) transmission rates. (a) Sagay et al. (2015) and Jamieson et al. (2012) did not provide a rate of transmission, but the rate was calculated on the basis of the number of children at risk provided in the paper. For Jamieson et al. (2012) transmission rate was provided at 28 weeks. For Ngoma et al. (2015) and Marazzi et al. (2009), a confidence interval was not provided in the paper but calculated using the formula^18^. (b) Coovadia et al. (2012), Alvarez-Uria et al. (2012), Thomas et al. (2011) and Kilewo et al. (2009) excluded positive children at six weeks. Jamieson et al. (2012) excluded transmission at two weeks and Marazzi et al. (2009) excluded four weeks. Jamieson et al. (2012), Thomas et al. (2011) and Kilewo et al. (2009) did not provide rate of transmission, but rate was calculated considering number of children at risk provided in the paper.

For six studies overall transmission rates (including peripartum) at age six months were provided [5,11,19,20,22,23] (Figure 2A).

In two studies ART was provided since 15 weeks of pregnancy [22,25]. Ngoma et al [25] reported three peripartum transmissions (before six weeks, number of children at risk = 219), and no postnatal transmissions between six weeks and six months, with an overall transmission rate at six months of 1.4% (95% CI 0.5–3.9%). Marazzi et al. [22] reported six transmissions at six months (N = 313, overall transmission rate 1.9%, 95% CI 0.9–4.1%). Sagay et al. [26] did not describe time for beginning of ART and a total of four infections was reported at six months (N = 856; rate of transmission was calculated as 0.5%, 95% CI 0.2–1.2%).

The subsequent studies started ART at later stages, Jamieson et al [19] started ART before 30 weeks pregnancy, and remaining studies started at 34 weeks pregnancy [20,23]. Jamieson et al. [19] reported 67 infections; 21 infections after six weeks (N = 849; overall transmission rate at age six months 7.9%, 95% CI 6.2–9.9%). Thomas et al. [20] reported 24 transmissions, of which 20 occurred before six weeks, for an overall transmission rate of 5.0%, 95% CI 3.4–7.4% (N = 487). Kilewo et al. [23] reported 22 infections at six months, 18 before six weeks (N = 423; overall transmission rate 5.0%, 95% CI 2.9–7.1%).

The pooled estimate of overall transmission at six months was 3.54% (95% CI 1.15–5.93%), with considerable heterogeneity (I^2^ 94.0%) (Figure 2A).

## Postnatal transmission between four and six weeks and six months

Six studies provided estimates of postnatal transmission rates, excluding peripartum infections, diagnosed before six weeks of age [5,11,19,20,22,23] (Figure 2B) and among those, three studies provided ART since the first antenatal visit [5,11,22]. Coovadia et al. [11] reported one infection (N = 413, rate 0.2%; 95% CI 0–1.4%); Marazzi et al. [22] provided the rate of transmission after four weeks of age (2/313, rate 0.6%; 95% CI 0.2–2.3%). Alvarez-Uria et al. [5] reported four (N = 127) infections for a transmission rate of 3.1% (95% CI 1.2–7.8%), noting that one of the infected infants was mixed fed.

In the other three studies, Jamieson et al. [19] reported 21 infections (N = 769, rate 2.7%; 95% CI 1.80–4.10%); Thomas et al. [20] and Kilewo et al. [23] did not provide a rate of postnatal transmission at six months, but the number of transmissions and number of children at risk were reported. In each study there were four transmissions between six weeks and six months of age (N = 482 for Thomas et al., rate of postnatal transmission 0.80%, 95% CI 0.3–2.4%; and N = 418 for Kilewo et al., rate of postnatal transmission 0.90%, 95% CI 0.3–2.1%). Figure 2B shows the pooled estimate for these six studies, with a pooled transmission rate of 1.08% (95% CI 0.32–1.85%). Heterogeneity was substantial (I^2^ 66.4%).

Two studies reported the number of infections at six months but not the number of children at risk. Thakwalakwa et al. [21] reported three infections before six months (of 280 births but no information provided on loss to follow up and deaths); Giuliano et al. [27] reported four transmissions before six months, 288 children were included in this study, but the number of children at risk of transmission at six months was unclear.

## Rate of transmission assessed after six months of age

**Figure 3.**
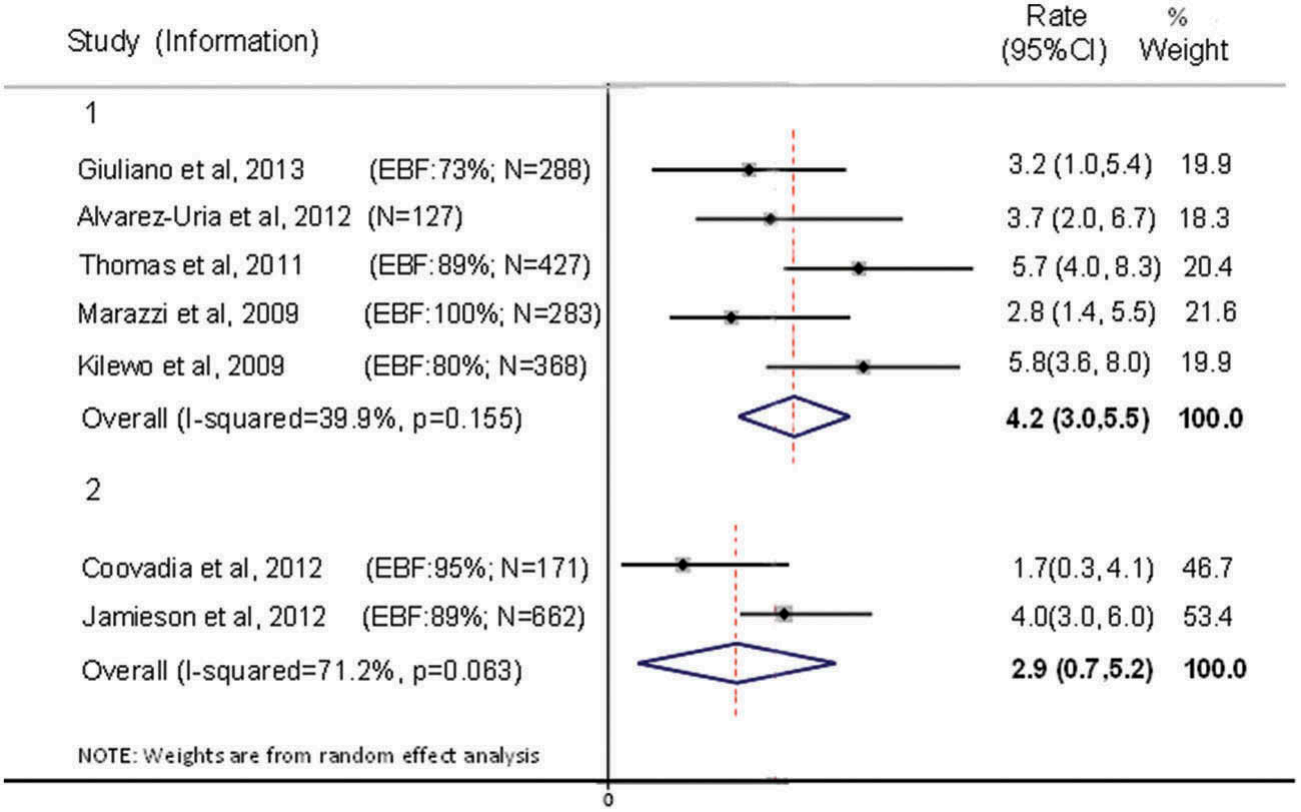
Transmission rates at 12 months of age, with 95% confidence intervals, in children who were breastfed and whose mothers were on ART. *Group 1*: Overall transmission. *Group 2*: Postnatal transmission between four and six weeks and 12 months of age. Note: Red dashed lines represent final pooled estimate for overall (a) and postnatal (b) transmission rates. Coovadia et al (2012) excluded children HIV positive at six weeks. Jamieson et al. (2012) excluded transmission at two weeks. Marazzi et al. (2009) did not provide the confidence interval, which was thus calculated using the formula^18^.

Of the seven studies providing information on transmission rates at age 12 months, five reported overall HIV transmission rates [5,20,22,23,27] and two reported postnatal transmission rates [11,19] (Figure 3). The pooled estimates showed an overall rate of transmission at 12 months of 4.2% (95% CI 2.97–5.5%); and a postnatal transmission rate of 2.93% (95% CI 0.68–5.18%) (Figure 3). Heterogeneity was higher in the postnatal transmission (I^2^ 71.2%) than in the overall HIV transmission group (I^2^ 39.9%). The postnatal pooled estimates included only two studies, one in which mothers initiated ART from first antenatal visit, and the other in which ART was initiated until 30 weeks.

Peltier et al. [24] provided both overall and postnatal transmission rate at nine months of age, with no transmission after cessation of breastfeeding. The total number of children at risk was 227, with four infections (only one after six weeks). The overall estimated rate of transmission at nine months was 1.8% (95%CI 0.7–4.8%), and postnatal 0.5% (95% CI 0.1–3.4%). Only 15 mothers were reported to have mixed fed their children, and none of these were infected. Kilewo et al [23] also provided rate of transmission at 9 months, in which 23 children were infected (N = 423), and five infections occurred after six weeks. Overall transmission was 5.3% (95% CI 3.2–7.4%) and postnatal transmission was 1.2% (95% CI 0.5–2.8%).

Four studies provided rate of transmission at 18 months, but those could not be pooled due to high heterogeneity. Ngoma et al. [25] was the only study which reported an estimated overall rate of transmission at 18 months, with lifelong ART provided for all mothers. Nine transmissions were reported, with an overall transmission rate of 4.1% (95% CI 2.2–7.6%; N = 219). Sagay et al. [26] also provided mothers with lifelong ART, but the study was a retrospective cohort using the same denominator for all rates, what does not allow for censoring due to loss to follow up or death. The study reported two additional transmissions after six months, and rate of transmission at 18 months was estimated as 0.7%. Kilewo et al. [23] and Thomas et al. [20] both provided overall transmission at 18 months, as 6.0% (95% CI 3.7–8.3%) and 6.7% (95% CI 4.8–9.4%) respectively. In both studies mothers started ART at 34 weeks gestation and stopped at six months postpartum.

## Additional information regarding feeding

No study provided a transmission rate according to infant feeding modality (EBF and MF); in all studies mothers were recommended to (and assumed to have) exclusively breastfed their infants for six months. Alvarez-Uria et al. [5] noted that one of the infected children was mixed-fed, but did not provide the rate of transmission by feeding modality.

Additional information was asked from each study regarding how feeding type was assessed and supported during the study, with data also extracted from studies (Table 1).

## Grade profile

An evaluation of the quality of the studies considered in this analysis is given in Supplementary Table 3. The assessment of quality was based on study limitations/risk of bias as per the evidence from the Newcastle-Ottawa Scale (Supplementary Table 4). We also considered inconsistency, indirectness and publication bias. All studies are rated very low quality. Initially, all studies were scored low quality due to being observational and were downgraded for indirectness because their research areas were not directly in line with the question. Where a pooled analysis was undertaken and a pooled estimate provided, studies were further downgraded for inconsistency if the heterogeneity was not explained. In all groups of studies there was at least one study with a risk of bias pertaining to lack of detailed information on feeding leading to further downgrading as did the substantial heterogeneity in transmission rate estimates between studies.

### Discussion

This systematic review is the first synthesis of the HIV transmission risk in infants of HIV-positive mothers including mothers receiving lifelong ART. The review provides evidence for the low postnatal HIV transmission risk to infants in the presence of maternal ART. We found a pooled estimated rate of overall transmission by age six months of 3.5% and a pooled postnatal transmission rate by six months of age of 1.1% in women who were on ART from early-mid pregnancy and who were recommended to breastfed their infants for six months. The pooled estimate for postnatal transmission at 12 months of age was 3.0%; only one study provided an estimated rate of transmission at 18 months, in women on lifelong ART, of 4.1%.

Our estimates compare favourably against estimates from studies in the pre-ART era, ranging from 15% overall transmission at six weeks [28] and 32% at six months [29,30], highlighting the efficacy of ART in reducing transmission risk following improved PMTCT services, adherence to ART, and early initiation during pregnancy [2,31].

Exclusive breastfeeding to six months of life is recommended for all infants because of its major effect on reducing infant and child mortality and improving long-term health outcomes. In the context of postnatal HIV transmission and before WHO guidelines recommending ART to reduce postnatal transmission, refraining from mixed-feeding in these first six months was also considered important [12,32] because of the associated increased risk of postnatal transmission when compared to EBF [33–35]. However, we were unable to estimate the rate of transmission associated with MF, since no study provided this data. Most studies included in this review assumed that mothers exclusively breastfed up to six months as recommended [6], however it has been reported by others that mixed feeding before six months of age is common [12,36]. Kilewo et al. [23] found that although at 16 weeks 80% of mothers were still exclusively breastfeeding, this was only 51% at 24 weeks; in the Kisumo Breastfeeding Study [20] exclusive breastfeeding at five months was 80.4%. EBF was found to be more common in women of higher parity, and less common in those not living with the child’s father [37]. In a qualitative study in Nigeria investigating reasons for mixed feeding, 42.8% of mothers reported pressure from family members relating to infant feeding practice, with 28.5% following cultural practices, offering water and herbal medications to the child [38].

In the included studies, women were recommended to exclusively breastfeed their infants for six months in line with WHO guidelines, but there was no specific support provided for mothers to exclusively breastfeed, and studies did not collect detailed information on infant feeding modality. As such, findings from this review thus reflect real-life situations. However, the reports from two studies included in this systematic analysis shows that support may increase adherence to exclusive breastfeeding. Marazzi et al. [22] offered nutritional supplements to pregnant and lactating women, and at each clinic visit, EBF counselling could be emphasised; Ngoma et al. sent text messages and offered food and transportation stipends to mothers participating in the study [25]. Both studies had very high adherence to EBF, with more than 90% of mothers reportedly exclusively breastfeeding during the first six months.

WHO 2010 guidelines on HIV and infant feeding recommend exclusive breastfeeding in the first six months of life primarily to protect against other non-HIV infectious causes of infant mortality. Based on the available evidence at that time, ART was assumed to be effective at reducing HIV transmission risk in this period. According to WHO guidelines, after six months, appropriate complementary food needs to be introduced while breastfeeding continues, although currently HIV-positive women are recommended to continue breastfeeding for one year under the cover of ART [6,8]. Only two studies followed the recommendation for HIV-positive women, with breastfeeding up to 12 months postpartum and lifelong ART to mothers [25,26]. In other studies mothers no longer received ART after cessation of breastfeeding at six months, but there were reported transmissions beyond six months due to continued breastfeeding in the absence of ART cover [19,27]. Despite providing lifelong ART and recommending breastfeeding up to 12 months, Ngoma et al. [25], reported high differences between transmission rate at six months (1.4%) and 18 months (4.1%) due to poor adherence among mothers at taking ART. They report that 11.4% of women who missed a dispensary visit transmitted HIV to the child, compared with 2.7% women who did not miss any visit (p = 0.038). On the other hand, the BREICHT study conducted in Malawi [21] found a very similar rate of transmission between six and 12 months (three infections before six months, four infections at 12 months, N = 280). This result could be attributed to high adherence to ART when it was given together with food supplements for the child after six months, a strategy that proved to be feasible and effective for improving retention in care.

This review highlighted the considerable clinical and methodological heterogeneity in the studies due to differences in the time of ART initiation during pregnancy, the recommended duration of breastfeeding, the age at which infection in the infant was assessed, the details on infant feeding modality and the definition of postnatal transmission. In addition, we calculated transmission rates and/or confidence intervals, where these were not provided by all studies, which contributed to the low quality of evidence for the estimated risk associated with continued breastfeeding between six and 12 months post-delivery. However, this systematic review and meta-analysis which includes studies published between 2005 and 2015 informs our understanding of the risk of transmission in the postnatal period in women who receive ART. Although statistical inconsistency was considerable, the trends were in the expected direction, and the findings provide evidence for public health programmes as well as for new MTCT guidelines.

#### Conclusion

The findings of this review demonstrate lower risk of postnatal transmission when women are on ART and breastfeeding, despite considerable heterogeneity in pooled analyses. The overall quality of this evidence is low. The new WHO guidelines on initiation of ART for all HIV-positive people immediately upon the diagnosis of HIV infection will expand ART coverage among HIV-positive pregnant and breastfeeding women [13]. Our results suggest that will be important to provide women with ongoing support to adhere to the WHO EBF recommendations.

## Notes

To access the supplementary material to this article please see Supplementary Files under Article Tools online.
